# *In Vivo* Cognitive-Enhancing, *Ex Vivo* Malondialdehyde-Lowering Activities and Phytochemical Profiles of Aqueous and Methanolic Stem Bark Extracts of *Piliostigma thonningii* (Schum.)

**DOI:** 10.1155/2020/1367075

**Published:** 2020-03-24

**Authors:** Gervason Apiri Moriasi, Anthony Muriithi Ireri, Mathew Piero Ngugi

**Affiliations:** ^1^Department of Biochemistry, Microbiology and Biotechnology, Kenyatta University, P.O BOX 43844-00100 Nairobi, Kenya; ^2^Department of Educational Psychology, Kenyatta University, P.O BOX 43844-00100 Nairobi, Kenya

## Abstract

Cognitive impairment (CI) is among the leading causes of disability in humans. It is estimated that over 35.6 million people are suffering from Alzheimer's disease- (AD-) associated cognitive deficits globally with these statistics projected to rise over 115.4 million by the year 2050. There is no specific etiology for this cognitive impairment; however, various contributing factors including advancing age (>60 years old), oxidative stress, cerebral injuries, infections, neurologic disorders, and cancer have been implicated. Despite various attempts to manage CI, no curative medicines are yet available. The current drugs used to manage symptoms of AD-associated CI including Donepezil and Rivastigmine among others are only palliative rather than therapeutic. Furthermore, these agents have been associated with undesirable side effects. This calls for alternative and complementary approaches aimed at either preventing or reverting AD-related CI in a curative way without causing adverse events. It is estimated that over 80% of the world's population utilize herbal medicines for basic healthcare as it is considered safe, affordable, and easily accessible as opposed to conventional healthcare. Various parts of *P. thonningii* are used in traditional medicine to manage various conditions including CI. However, empirical and scientific data to validate these uses is lacking. In this study, the Morris water maze (MWM) experiment was adopted to evaluate the cognitive-enhancing effects of the studied plant extracts. The malondialdehyde (MDA) profiles in the brains of experimental mice were determined using the thiobarbituric acid reactive substances (TBARS) test. Moreover, qualitative phytochemical profiling of the studied plant extracts was performed using standard procedures. The results showed remarkable cognitive-enhancing activities which were reflected in significantly shorter transfer latencies, navigation distances, longer time spent in platform quadrant, and lower MDA levels compared with those recorded for the negative control mice (*p* < 0.05). Phytochemical screening of the studied plant extracts revealed the presence of antioxidant phytocompounds, which may have played key roles in the extracts' potency. Based on the findings herein, *P. thonningii* extracts, especially the aqueous ones have a promising potential for the management of AD-associated CI. Further studies aimed at isolating and characterizing specific active compounds for CI from *P. thonningii* are recommended. Additionally, specific mode(s) of action of active principles should be elucidated. Moreover, toxicity studies should be done on the studied plant extracts to ascertain their safety.

## 1. Introduction

Cognitive impairment is the overarching phenomenon referring to a continuum of diseases and mental impairments of the brain tissue, which cause abnormalities in learning, memory, and communicative and intellectual abilities of the affected subject. Cognitive impairment is a complex presentation of continuous degeneration of intellectual functions characterized by a plethora of symptoms caused by diseases/disorders affecting the brain [1, 2]. It is estimated that 35.6 million people are affected with AD and associated cognitive deficits, with over half (58%) living in low- and middle-income countries. Annually, 7.7 million new cases are reported and these figures are expected to almost double every 20 years, to 65.7 million in 2030 and 115.4 million in 2050 [3].

Cognitive impairment is one of the major causes of mental disability and dependency among older people and those suffering from AD and other dementias globally. It is not only debilitating to the affected subjects but also to their caregivers and families. There is often a lack of awareness and understanding of cognitive disorders, resulting in stigmatization and barriers to diagnosis, treatment, and management. The impact of cognitive impairment on caregivers, family, and society in general can be physical and psychological [4].

Recent research has shown that oxidative stress, which is the excessive generation of free radicals or their ineffective attenuation, drives cognitive impairment by damaging brain cells [5]. The high concentration of polyunsaturated fatty acids (PUFAs) in the brain increases its vulnerability to free radicals attack, which, in turn, damage brain cells leading to CI [6, 7]. Lipid peroxidation is thought to be a destructive form of oxidative degradation that impairs cell membranes, thereby generating several secondary products including reactive oxygen metabolites (ROMs) that cause neurotoxicity [8]. An increase in the levels of malondialdehyde (MDA), which is one of the ROMs, has been recognized as an important lipid peroxidation indicator and a marker of oxidative stress [9, 10].

Oxidative damage to proteins causes impairment to endogenous enzymes, which play important roles in proper functioning of the neural and glial cells in the brain. Damage to critical enzymes in the brain may lead to the downregulation of energy metabolism, which in turn triggers cell damage. Moreover, protein oxidation in the central nervous system exacerbates hyperphosphorylation of the tau proteins with aggregations of *β*-amyloids, which are hallmark features in cognitive-impaired brains of AD-affected patients [11, 12].

Even though pharmacotherapies seem to slow cognitive impairment events, the advantages are mostly marginal and unreliable [13]. Conventional medicines prescribed for the management of cognitive impairment are only palliative and do not cure the disease as they only manage the symptoms and improve quality of life without altering the result of the underlying condition [13, 14]. Furthermore, due to the diverse constellation of neuropsychiatric and behavioral symptoms associated with cognitive impairment, there may be potential undesirable side effects of conventional drugs, where improving one symptom worsens another symptom, thus rendering the management of this condition an uphill task. As a result, there is an urgent need for arsenal therapeutic agents for the management of AD-associated CI, and medicinal plants' phytocompounds are a potential source [12, 15–17].

Plants have been used since antiquity for the management of cognitive deficits [18–20]. Various plant secondary metabolites that are responsible for their biological activities have been identified [21]. Some of them include terpenoids, phenolic compounds (flavonoids, phenolic acids, quinones, coumarins, lignans, stilbenes, and tannins), nitrogen-containing phytocompounds (alkaloids, amines, and betalains), and carotenoids. Phenolics have been demonstrated to possess the widest spectrum of bioactivity by acting as potent antioxidants [16, 21, 22]. The beneficial properties exerted by these bioactive components of medicinal plants include the inhibition of acetylcholinesterase (AChE), modification of A*β* processing, protection against apoptosis, and attenuation of oxidative stress [12, 15, 16]. Therefore, utilization of antioxidants offers a promising potential in the prevention and treatment of AD-associated cognitive deficits as well as other associated neurologic conditions [5, 8, 23].

Various parts of *P. thonningii* are used in African traditional medicine for the treatment of diverse conditions and diseases. Briefly, *P. thonningii* is used for the treatment of malaria, leprosy, ulcers, fever, wounds, arthritis, dizziness, coughs, and dementia and memory enhancement among others [24]. Some of the isolated bioactive compounds from *P. thonningii* include D-3-*O-*methylchiroinosital which possess antioxidant, analgesic, antidiabetic, antilipidemic, and antipyretic activities. Additionally, C-methyl flavanols, which have anti-inflammatory and antibacterial activities, have been isolated from this plant [25]. Furthermore, other antioxidant compounds with broad spectra of activities like *β*-amyrin **(7)** [25], quercetin **(11)**, and quercitrin **(12)** [26], among many others **(1-6, 8-10,** and **13-15)**, have so far been isolated and identified [25]. Therefore, this study was aimed at investigating *in vivo* cognitive enhancing, *ex-vivo* MDA profile lowering and qualitative phytochemical composition of the aqueous and methanolic stem bark extracts of *P. thonningii* in the quest for better, safe, cost-effective, and potent novel drugs for the treatment of AD-associated cognitive deficits.

## 2. Materials and Methods

### 2.1. Plant Material and Processing

Fresh stem barks of *P. thonningii* were collected from Cianyi village, Muchomoke sublocation, Gitiburi location, Siakago division, Mbeere North Subcounty in Embu County, Kenya, where the plant grew naturally. This plant was chosen for this study based on its ethnomedical information and use among the locals. The plant was identified primarily by its local name (“*Mukuura*”) and diseases it treats by the help of a reputable local herbalist. Voucher specimen was prepared, identified, and authenticated (GM001/2017) by a taxonomist at the Department of Plant Sciences, Kenyatta University, and the specimen was deposited for future reference. The collected barks of the plant were then cut into small pieces and spread evenly to dry under shade at room temperature for a period of 14 days. Regular grabbling was done to ensure uniform drying. The dried material was then ground into a coarse powder with the help of an electric plant mill and stored in a well-labeled khaki envelope awaiting extraction.

### 2.2. Preparation of Methanolic and Aqueous Extracts

Approximately 200 grams of the powdered material- of *P. thonningii* barks were macerated in 750 ml of methanol (AR grade) in 1-liter conical flask, with regular shaking for 48 hours. The methanolic mixture was carefully decanted and filtered through Whatman No. 1 filter paper. The filtrates were concentrated *in vacuo* with the help of a rotary evaporator set at 50°C, transferred into preweighed, clean, dry, and labeled universal glass bottles. They were then kept in a hot-air oven set at 35°C for 5 days to allow for complete drying. The aqueous extract was obtained by boiling 50 g of powdered *P. thonningii* bark in distilled water for five minutes. The extracts were then cooled to room temperature, filtered, and transferred into clean freeze-drying flasks. The flasks were then fitted into a freeze dryer for lyophilization for a period of 48 hours. The dry and lyophilized extracts were transferred into clean, dry, preweighed, and labeled universal glass bottles. The actual weights of the extracts were calculated by subtracting the weight of the empty bottle from that of the bottle containing the extract. The percentage yields of respective extracts were calculated using the formula described by Harborne (1976) and modified by Afolayan et al. [25] and Truong et al. [27]. The respective extracts were then sealed and stored in a refrigerator at 4°C awaiting biological and chemical assays.

(1) $$\begin{array}{c} \%\text{Yield}=\frac{\text{Weight of extract}}{\text{Weight of macerated powder}}\times 100. \end{array}$$

### 2.3. Determination of *In Vivo* Cognitive-Enhancing Effects

#### 2.3.1. Experimental Animals

In this study, Swiss-Albino mice (white) aged 4-5 weeks with an average weight of 24 ± 2 g were obtained from the animal breeding house of Kenya Medical Research Institute (KEMRI) Nairobi, Kenya. The mice were kept in standard conditions and housed in polypropylene rectangular cages measuring 30 cm × 20 cm × 13 cm with soft wood shavings as bedding material. They were randomly selected and housed in groups of 3 males and 2 females in separate home cages, provided with standard laboratory animal food (rodent pellets) and tap water *ad libitum*. They were maintained at a natural 12-hour-day/12-hour-night cycle in a room maintained at 24 ± 1°C, 360 lux lighting, and 65% humidity. They were acclimatized for 72 hours before experimentation. Humane handling and standard protocols/guidelines for laboratory animal care and use were followed in this study according to the National Research Council [25], after Kenyatta University ethical approval and National Commission for Science, Technology and Innovation authorization (NACOSTI/P/19/2080).

#### 2.3.2. Preparation of Drugs and Administration

The methanolic and aqueous stem bark extracts of *P. thonningii* for administration at dose levels of 200 mg/kg bw, 100 mg/kg bw, and 50 mg/kg bw were prepared freshly each day in normal saline according to the guidelines described by [26]. The positive control (Donepezil) was also prepared at a dose level of 1 mg/kg bw in normal saline using the same guidelines. All the drugs were orally administered (*p.o*) to respective experimental groups of mice at 0900 hrs during the experimentation period. Hyoscine hydrobromide (scopolamine) was prepared in normal saline at a dose of 1 mg/kg bw in the same manner as for extracts and Donepezil. It was administered intraperitoneally (*i.p*) during the experiment.

#### 2.3.3. Morris Water Maze Setup

The Morris water maze method described by Morris [27–29] was used in this study for determination of *in vivo* cognitive-enhancing effects of the two medicinal plant extracts. The water maze comprised a white circular tank that formed a pool measuring 110 cm in diameter and 45 cm in height with a featureless inner surface. The circular pool was filled with water, in which 750 g of powdered fat-free milk was mixed, to a height of 30 cm to make the pool opaque. The temperature of the maze was monitored using a calibrated mercury bulb thermometer and maintained at 26 ± 1°C. A white escape platform measuring 10 cm in diameter and 29 cm in height was centered in the northwest (NW) quadrant of the pool. The water level was adjusted to 1 cm below the top surface of the escape platform and later to 1 cm above the escape platform. On the walls of the maze, manila papers of blue, green, pink, and yellow colors were mounted to the west (W), north (N), south (S), and east (E) quadrants, respectively, as local visual cues before introducing the mice. The continuous location of each swimming mouse, from the start position to the top of the platform, was monitored with the help of a digital Sony video camera that was mounted 1.5 meters above the maze linked to Any-Maze tracking software version 6.05 installed in Windows 10 Pro PC [27–29].

#### 2.3.4. Swim Training

In this study, the day prior to the experimentation day was dedicated to training each of the experimental mice to swim for 60 seconds in the presence of the visible escape platform followed by another training session in the absence of the escape platform, with an intertraining break of 20 minutes to allow the mice to rest and recover. This was followed by another training session with the invisible platform in the same manner as the preceding trainings, where each mouse could explore the maze, searching for the hidden platform so as to help it create a spatial map of the surroundings and for proper orientation. The location of the escape platform was in the northwest (NW) quadrant and remained unchanged throughout the training session. The specific starting points were predetermined (the boundaries of each quadrant) as north (N), south (S), east (E), and west (W), respectively, and were changed in every trial and session of experiment.

#### 2.3.5. Acquisition

In the acquisition days, the water level in the maze was adjusted to 1 cm above the escape platform, which was centered in the northwest (NW) quadrant to make it invisible at water level. Mice were subjected to three sessions each day for three consecutive days with an intertrial break of 20 minutes. The starting points were predetermined in the same manner as during the training session and were alternated throughout the experiment.

During each trial, mice were held and gently placed in the water maze facing the wall away from the escape platform. Each mouse could explore the pool, searching for the hidden escape platform for a maximum period of 60 seconds. Once the mouse located the platform, it could remain on it for 10 seconds for further examination of the surroundings. If the mouse did not locate the platform within 60 seconds, it was gently guided to the platform and allowed to rest there for 15 seconds as it explored the surroundings. It was then gently removed from the pool and placed in a holding cage that contained paper towels to dry. This parameter was averaged for each session of trials and for each experimental mouse. Transfer latency (time taken to search for and locate the hidden platform with 60 s cutoff) and the navigation distance (distance covered during the MWM task) for each experimental mouse were recorded by a digital video recorder.

#### 2.3.6. Probe Trial

To assess learning and memory retention, the experimental mice were subjected to a single probe trial on the last day of experimentation (Day 4) by introducing each of them into the maze without the escape platform. This helped assess whether the experimental mice had learnt the task and if they were able to remember the location of the hidden escape platform. They were allowed to explore and search for the escape platform as during the acquisition phase, and the respective times spent in the NW quadrant for each mouse were recorded.

#### 2.3.7. Induction of Cognitive Impairment

Cognitive impairment was induced during the last day of experimentation (Day 4) by an intraperitoneal (*i.p*) injection of 200 *μ*l of hyoscine hydrobromide (scopolamine) at a dose of 1 mg/kg bw to all the experimental mice except for the normal control mice, to which 200 *μ*l of normal saline was administered intraperitoneally.

#### 2.3.8. Experimental Design

A controlled randomized, laboratory-based study design was adopted, from which an experimental design was drawn. For each of the studied plant extracts, thirty (30) experimental mice were randomly assigned to six treatment groups each consisting of five mice (3 males and 2 females). The grouping protocol was as presented in Table 1.

All mice were subjected to the water maze task 30 minutes after cognitive impairment and allowed to navigate and search for the hidden platform for a maximum period of 60 seconds. They were observed and monitored in the same way as during the acquisition (Section 2.3.5) phase. The respective video clips recorded during each task trial performed by the mice were uploaded into Any-Maze version 6.05 software from which quantitative data were derived for statistical analysis [28, 29].

### 2.4. *Ex Vivo* Determination of Effects of the Extracts on Lipid Peroxidation

In this study, Thiobarbituric Acid Reactive Substances (TBARS) assay was used to determine the effects of the extracts on lipid peroxidation. In this experiment, the levels of the lipid peroxidation biomarker (malondialdehyde (MDA)) were measured. Following the Morris water maze test, all the mice were decapitated by cervical dislocation and their whole brains rapidly dissected under standard conditions, cleaned with ice-cold saline, and stored at -20°C. Brain tissue samples were thawed and homogenized with 10 ml of ice-cold 0.1 M phosphate buffer (pH 7.4). The reaction mixtures contained 1.5 ml of 20% acetic acid (pH 3.5), 1.5 ml of 0.8% thiobarbituric acid, 0.2 ml of 8.1% sodium dodecyl sulphate, and 0.1 ml of processed brain tissue samples. The mixtures were then heated at 100°C for 60 minutes, cooled, and 5 ml of n-butanol/pyridine (15 : 1) and 1 ml of distilled water added. The mixtures were vortexed vigorously for the contents to mix. The resultant mixtures were subjected to centrifugation at 2,500 rpm for 20 minutes, the organic layer was separated, and absorbance measured at *λ*_532_ nm using a UV-Vis spectrophotometer (Shimadzu UV-Vis 1600). The concentration of MDA was calculated using a molar extinction coefficient of 1.56 × 10^5^ M^−1^ cm^−1^ and expressed as *μ*mol/g of brain tissue [30, 31].

### 2.5. Qualitative Phytochemical Screening

Qualitative tests for various phytochemicals present in the aqueous and methanolic extracts of *P. thonningii* stem barks were carried out using standard phytochemical screening procedures. Visual examination of the appearance of color or frothing was used as an indicator for the presence or absence of a given phytochemical group.

#### 2.5.1. Test for Tannins

About 0.5 g of aqueous and methanolic extracts of *P. thonningii* were separately boiled with 5 ml of distilled water, respectively, in test tubes and then filtered. To the resultant filtrates, 3 drops of 0.1% FeCl_3_ were added. The appearance of blue-green precipitate indicates the presence of tannins [32].

#### 2.5.2. Test for Saponins (Frothing Method)

About 0.5 g of aqueous and methanolic extracts of *P. thonningii* were separately boiled with 5 ml of distilled water, respectively, in test tubes and then allowed to cool. After shaking, the appearance of frothing that persists for more than 2 minutes is indicative of the presence of saponins [30].

#### 2.5.3. Test for Alkaloids

Approximately 0.1 g of aqueous and methanolic stem bark extracts of *P. thonningii* were separately mixed with about 5 ml of 1% HCl respectfully, warmed, and filtered. 2 ml of filtrates was treated with Mayer's reagent. The appearance of cream-colored precipitate is a positive indication for the presence of alkaloids. Similarly, 2 ml of the filtered crude extracts was treated with Dragendorff's reagent. A reddish-brown precipitate indicates a positive test for the presence of alkaloids [31].

#### 2.5.4. Test for Glycosides

*(i) Keller-Killiani Test (Test for Deoxy Sugars)*. Approximately 0.2 g of aqueous and methanolic extracts were separately extracted with 5 ml of chloroform and evaporated to dryness in clean test tubes. 0.4 ml of glacial acetic acid containing trace amount of ferric chloride was added followed by careful addition of 0.5 ml of concentrated sulphuric acid by the side of the test tube. Acetic acid layer showing a blue color is a positive test for the presence of cardiac glycosides [33].

*(ii) Borntrager's Test*. About 0.1 grams of studied extracts were boiled separately with 1 ml of sulphuric acid in test tubes for 5 minutes. They were then filtered while hot, cooled, and then shaken with equal volume of chloroform. The lower layers of chloroform were separated and shaken with half of their volumes with dilute ammonia. A rose pink to red color produced in the ammoniacal layer is a positive indication of the presence of glycosides [34].

*(iii) Modified Borntrager's Test*. Two drops of Kedde reagent were added to portions of studied extracts (0.1 g). The presence of purple color indicates the presence of glycosides whose aglycone moiety has unsaturated lactone ring [34].

#### 2.5.5. Test for Steroids

To 1 ml solutions of extracts, 3 drops of the Liebermann–Burchard reagent were added. The production of a reddish-purple color indicates the presence of steroids [34].

#### 2.5.6. Test for Flavonoids

Five drops of concentrated hydrochloric acid were added to 1 ml of alcoholic extracts of the test plant materials. Immediate development of a red color indicates the presence of flavonoids. The presence of flavonoids was also confirmed by three other methods. 10 ml solutions of the test extracts were hydrolyzed with 10% sulphuric acid and divided into three portions. To the first portion, 1 ml dilute ammonia solution was added. Greenish-yellow color indicates the presence of flavonoids. To the second portion, 1 ml dilute sodium carbonate solution was added. Pale yellow indicates the presence of flavonoids. To the third portion, 1 ml of 10% sodium hydroxide solution was added. A yellow color indicates the presence of flavonoids [34].

#### 2.5.7. Test for Phenols

About 0.1 g portions of extracts were separately boiled with 10 ml of 70% ethanol in a water bath using boiling tubes for 5 minutes. The extracts were filtered through Whatman No. 1 filter papers while hot and carefully cooled under gently running tap water. To 2 ml of the respective extract filtrates, 5 drops of 5% ferric chloride were added. The occurrence of a green precipitate indicates the presence of phenols [34, 35].

#### 2.5.8. Test for Terpenoids

To about 2 ml of the test extracts, 5 drops of acetic acid anhydride were added. This was followed by careful addition of 5 drops of concentrated sulphuric acid. The formation of a blue-green ring indicates the presence of terpenoids [34].

#### 2.5.9. Test for Coumarins

About 0.2 g of the test extracts were warmed with 2 ml of ethanol. The Whatman filter paper treated with 10% NH_4_OH solution were placed on the mouth of the test tube for 5 minutes. Thereafter, the filter paper was illuminated with UV light (365 nm) and fluorescence observed. Yellow fluorescence indicates the presence of coumarins [35].

### 2.6. Data Management and Statistical Analysis

The yields of the crude drugs following extraction were expressed as percentages of total materials macerated. Quantitative data from Morris water maze (derived from the Any-Maze software v 6.05) and TBARS (MDA) profile assays were tabulated on a spreadsheet and then exported to Minitab v19.1 (State College, Pennsylvania). The data were subjected to descriptive statistics, expressed as mean ± SEM. One-Way ANOVA was used to test for statistical significance among the negative control (normal saline+scopolamine), positive control (Donepezil+scopolamine), and experimental groups (*P. thonningii* extracts of tested concentrations+scopolamine) at 95% confidence level. Furthermore, the data were subjected to Fisher's LSD *post hoc* test for pairwise comparison and separation of means. Comparisons between the activities of the two independent extracts of the studied plant on assayed parameters were performed using the unpaired Student *t*-test statistics. Values of *p* < 0.05 were considered statistically significant. Quantitative data were presented in the form of graphs and tables while qualitative data from phytochemical screening were tabulated.

## 3. Results

### 3.1. Percentage Yields of Extracts

Following extraction, the respective percentage yields of the studied plant extracts were determined. In general, relatively higher yields were obtained for the methanolic stem bark extract of *P. thonningii* compared with the aqueous counterpart as shown in Table 2.

### 3.2. *In Vivo* Cognitive-Enhancing Effects of Stem Bark Extracts of *P. thonningii*

To investigate the effects of the studied plant extracts on cognitive enhancement, the Morris water maze test for spatial memory was adopted. During the acquisition period, the transfer latencies and navigation distances were recorded.

#### 3.2.1. *In Vivo* Effects of the Aqueous and Methanolic Stem Bark Extracts of *P. thonningii* on Transfer Latency

*(i) During the acquisition phase(Days 1-3)*. The results showed that, in general, the mouse groups treated with the studied dose levels of aqueous stem bark extract of *P. thonningii* took significantly shorter transfer latency in each of the acquisition days (1-3) compared with the rest of the mouse groups (*p* < 0.05; Table 3). Notably, the mice that received the methanolic stem bark extract of *P. thonningii* at a dose level of 50 mg/kg bw took transfer latencies and navigation distances that were not significantly different from those taken by the negative control mice throughout the acquisition period (*p* > 0.05; Table 3). In Day 2 and Day 3, the group of experimental mice that received 200 mg/kg bw of aqueous stem bark extract of *P. thonningii* took the shortest time to complete the MWM task compared with the other groups (*p* < 0.05; Table 3).

**Figure 1:**
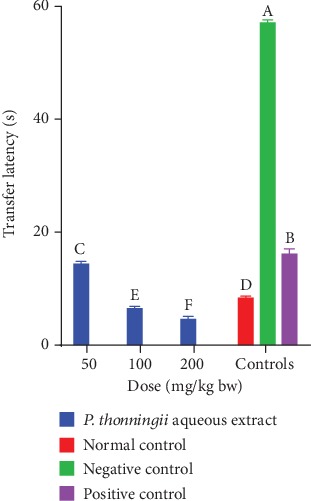
Effect of the aqueous stem bark extract of *P. thonningii* on transfer latency. Bars that do not share a letter are significantly different (one-way ANOVA followed by Fisher's LSD; *p* < 0.05).

*(ii) After scopolamine-induced cognitive impairment (Day 4)*. In the last day of experimentation, the experimental mice were cognitively impaired by scopolamine before performing the MWM task. The transfer latency in experimental mice treated with the aqueous stem bark extract of *P. thonningii* was significantly lower compared with the transfer latency of the mice in the positive and negative control groups at all the studied doses (*p* < 0.05; Figure 1). Moreover, the mice that were treated with the aqueous stem bark extract of *P. thonningii* at a dose of 50 mg/kg bw took significantly shorter transfer latency compared with the mice in the negative and positive control groups (*p* < 0.05; Figure 1). Furthermore, the experimental mice treated with the aqueous extract at doses of 100 mg/kg bw and 200 mg/kg bw took significantly shorter transfer latencies to complete the Morris water maze task compared with the mice in the positive and normal control groups (*p* < 0.05; Figure 1). Generally, dose-dependent reductions in transfer latencies were observed in experimental mouse groups into which aqueous stem bark extracts of the studied plants were administered (Figure 1).

**Figure 2:**
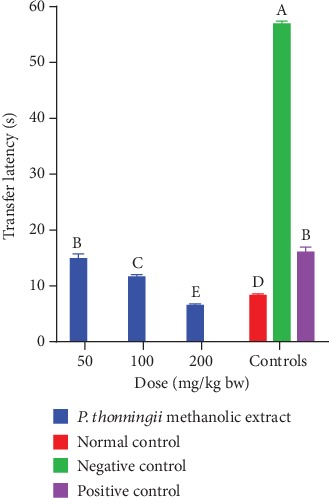
Effects of the methanolic stem bark extract of *P. thonningii* on transfer latency. Bars that do not share a letter are significantly different (one-way ANOVA followed by Fisher's LSD; *p* < 0.05).

Moreover, a dose-dependent reduction in transfer latency in cognitively impaired mice models treated with the methanolic stem bark extract of *P. thonningii* was observed (Figure 2). The transfer latency recorded by the experimental mice treated with the methanolic stem bark extract of *P. thonningii* at a dose of 50 mg/kg bw was not significantly different from the transfer latency recorded by the mice in the positive control group (*p* > 0.05; Figure 2).

At the extract doses of 100 mg/kg bw, there were significantly shorter transfer latencies in experimental mice than the transfer latency taken by the mice in the negative and normal control groups (*p* < 0.05; Figure 2). Notably, the mice administered with *P. thonningii* methanolic extract at a dose level of 200 mg/kg bw took significantly shorter time to complete the Morris water maze task compared with the mice in the normal and positive and negative control groups (*p* < 0.05; Figure 2).

**Figure 3:**
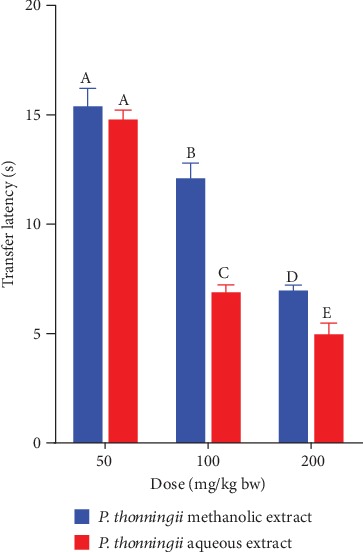
Comparison of the effects of the aqueous and methanolic stem bark extracts of *P. thonningii* on transfer latency. Bars with the same letter within the same dose level are not significantly different (*p* > 0.05) while those with different letters within the same dose level are significantly different (*p* < 0.05) (unpaired Student's *t*-test).

A comparison between the effects of the two studied plant extracts was done. The results showed that, at the extract dose of 50 mg/kg bw, the differences in the effects of the aqueous and methanolic stem bark extracts of *P. thonningii* on transfer latency in the experimental mice were not significant (*p* > 0.05; Figure 3). However, at doses of 100 mg/kg bw and 200 mg/kg bw, the mice treated with the aqueous stem bark extract of *P. thonningii* took significantly shorter transfer latency compared with the mice that were administered with the methanolic extract of *P. thonningii* at the same dose levels (*p* < 0.05; Figure 3).

#### 3.2.2. *In Vivo* Effects of the Aqueous and Methanolic Stem Bark Extracts of *P. thonningii* on Navigation Distance

The total distance covered by each mouse from the starting point to the escape platform was recorded in this study.

*(i) During the acquisition phase*. Generally, the results revealed that the mice that received the studied plant extracts at all dose levels except those that received 50 mg/kg bw of the methanolic extract covered significantly shorter distances in the MWM task throughout the acquisition period (*p* < 0.05; Table 4). During Day 3 of the acquisition trials, no significant differences in navigation distances were observed among experimental mice in the normal, positive, and the extract-treated (methanolic extract dose of 200 mg/kg bw and all the three doses of aqueous extract) groups (*p* > 0.05; Table 4). Furthermore, no significant differences in navigation distances were observed among mice in the negative control group and those that were administered with the methanolic stem bark extract of *P. thonningii* at dose levels of 50 mg/kg bw and 100 mg/kg bw (*p* > 0.05; Table 4).

Overall, significant reductions in navigation distances were observed in experimental mice with higher reductions being recorded for the extract-treated and positive control groups of mice (Table 4).

**Figure 4:**
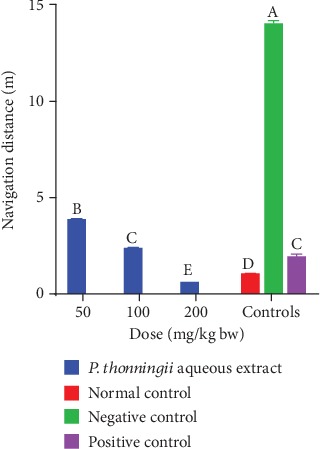
Effects of the aqueous stem bark extracts of *P. thonningii* on navigation distance. Bars with different letters are significantly different (one-way ANOVA followed by Fisher's LSD; *p* < 0.05).

*(ii) After scopolamine-induced cognitive impairment (Day 4)*. It was observed that the cognitively impaired mice into which a 50 mg/kg bw dose of the aqueous stem bark extract of *P. thonningii* was administered covered significantly shorter distance to reach the escape platform compared with the mice in the negative control group (*p* < 0.05; Figure 4). However, it was noted that this group of mice covered significantly longer distances to complete the task compared with the positive control mice (*p* < 0.05).

At the dose of 100 mg/kg bw, there was no significant difference in the navigation distance covered by the mice treated with the aqueous stem bark extract of *P. thonningii* and that covered by the mice in the normal control group (*p* > 0.05; Figure 4). A significantly shorter navigation distance was covered by the mice administered with the *P. thonningii* extract dose of 200 mg/kg bw compared with the distances covered by the positive, normal, and negative control groups of mice (*p* < 0.05; Figure 4).

**Figure 5:**
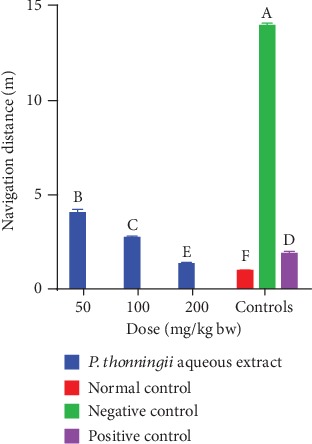
Effects of the methanolic stem bark extracts of *P. thonningii* on navigation distance. Bars with different letters are significantly different (one-way ANOVA followed by Fisher's LSD; *p* < 0.05).

The experimental mice that were treated with the methanolic stem bark extract of *P. thonningii* at dose levels of 50 mg/kg bw and 100 mg/kg bw covered significantly shorter distances to find the escape platform compared with the mice in the negative control group (*p* < 0.05; Figure 5). However, at these dose levels, the treated mice covered longer distances compared with the positive control mice. A significantly shorter navigation distance was covered by the mice that were treated with the methanolic stem bark extract of *P. thonningii* at a dose of 200 mg/kg bw compared with the distance covered by the mice in the positive control group (*p* < 0.05; Figure 5).

**Figure 6:**
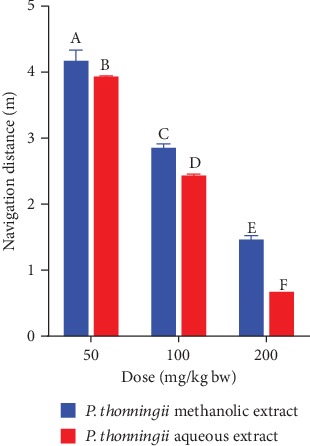
Effects of the aqueous and methanolic stem bark extracts of *P. thonningii* on navigation distance. Bars with different letters within the same dose level are significantly different (unpaired *t*-test at 95% confidence level; *p* < 0.05).

A comparison of the effects of the studied plant extracts on navigation distance was done. There was a significant difference between the navigation distances of experimental mice that were administered with the studied plant extracts at a dose level of 50 mg/kg bw (*p* < 0.05; Figure 6). The results also revealed that the experimental mice that were treated with dose levels of 100 mg/kg bw and 200 mg/kg bw of the aqueous stem bark extract of *P. thonningii* covered significantly shorter distances compared with the mice that received similar doses of the methanolic stem bark extract of *P. thonningii* (*p* < 0.05; Figure 6).

#### 3.2.3. Spatial Memory Retention (Probe Trial: Day 4)

**Figure 7:**
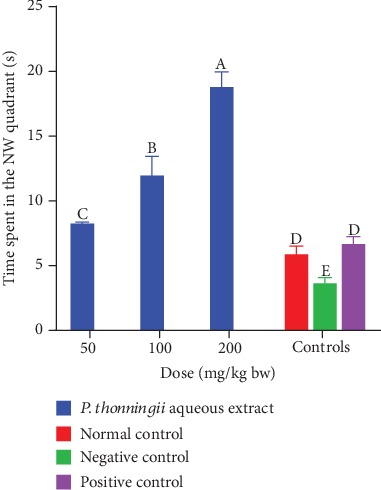
Effects of the aqueous stem bark extracts of *P. thonningii* on spatial memory retention. Bars with different letters are significantly different (*p* < 0.05) while those sharing a letter are not significantly different (*p* > 0.05) (one-way ANOVA followed by Fisher's LSD).

The results revealed that the cognitive-impaired mice which were treated with the aqueous stem bark extracts of *P. thonningii* at all the three dose levels spent significantly more time in the NW quadrant, where the escape platform was previously located, compared with the mice in all the control groups (*p* < 0.05; Figure 7). Generally, a dose-dependent increase in the time spent in the NW quadrant was noted in mice that were administered with the aqueous extract (Figure 7).

**Figure 8:**
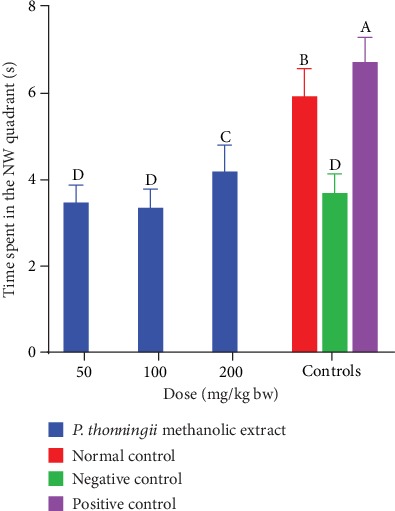
Effects of the methanolic stem bark extract of *P. thonningii* on spatial memory retention. Bars with the same letter are not significantly different (*p* > 0.5) while those with different letters are significantly different (*p* < 0.05) (one-way ANOVA followed by Fisher's LSD).

On the other hand, results revealed that mice which received the methanolic stem bark extract of *P. thonningii* at a dose of 200 mg/kg bw spent significantly more time in the NW quadrant compared with the negative control mice in the same quadrant (*p* < 0.05; Figure 8). However, there was no significant difference in the time spent in the NW quadrant among the mice that were treated with the methanolic extract at dose levels of 50 mg/kg bw and 100 mg/kg bw and those in the negative control group (*p* > 0.05; Figure 8).

**Figure 9:**
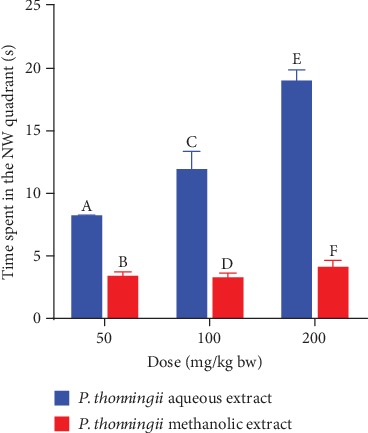
Effects of the aqueous and methanolic stem bark extracts of *P. thonningii* on spatial memory retention. Bars with different letters within the same dose level are significantly different (unpaired *t*-test at 95% confidence level; *p* < 0.05).

A comparison between the effects of the two studied plant extracts on spatial memory retention was also done in this study. The results showed that the experimental mice that were administered with the aqueous stem bark extract of *P. thonningii* at the three studied dose levels spent remarkably more time in the NW quadrant compared with the mice that received the methanolic extract at similar dose levels (*p* < 0.05; Figure 9).

### 3.3. *Ex Vivo* Effects of the Aqueous and Methanolic Stem Bark Extracts of *P. thonningii* on MDA Profiles

**Figure 10:**
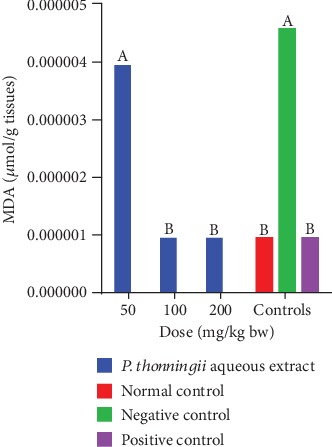
Effects of the aqueous stem bark extract of *P. thonningii* on MDA profile. Bars with the same letter are not significantly different (one-way ANOVA followed by Fisher's LSD; *p* > 0.05).

The results showed no significant difference in MDA levels in the brain of mice that were treated with a dose of 50 mg/kg bw of the aqueous stem bark extract of *P. thonningii* compared with the MDA levels in the brains of the mice in the negative control group (*p* > 0.05; Figure 10). However, the mice that were administered with the *P. thonningii* extract doses of 100 mg/kg bw and 200 mg/kg bw had significantly lower MDA profiles in their brains compared with the mice in the negative control group (*p* < 0.05; Figure 10). Moreover, the MDA profiles in the brains of experimental mice treated with the *P. thonningii* extract doses of 100 mg/kg bw and 200 mg/kg bw were not significantly different from the MDA profiles in the brains of the mice in the positive and normal control groups (*p* > 0.05; Figure 10).

**Figure 11:**
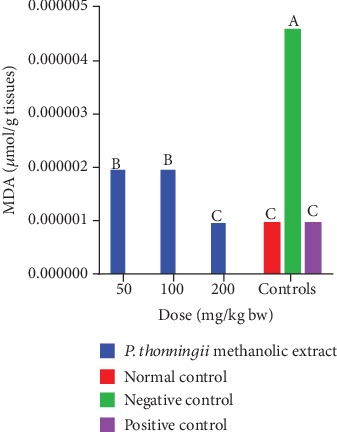
Effects of the methanolic stem bark extract of *P. thonningii* on MDA profile. Bars with the same letter are not significantly different (*p* > 0.05) while those with different letters are significantly different (*p* < 0.05) (one-way ANOVA followed by Fisher's LSD).

In general, the results showed that the mice that were treated with the methanolic stem bark extract of *P. thonningii* had significantly lower MDA profiles in their brains compared with the mice in the negative control group (*p* < 0.05; Figure 11). There were no significant differences in MDA levels in the brains of mice that were treated with the methanolic stem bark extract of *P. thonningii* at doses of 50 mg/kg bw and 100 mg/kg bw (*p* > 0.05; Figure 11). However, at a dose of 200 mg/kg bw, the MDA profile in the brains of the experimental mice that received the methanolic stem bark extract of *P. thonningii* was not significantly different from the MDA levels in the brains of mice in the normal and positive control groups (*p* > 0.05; Figure 11).

**Figure 12:**
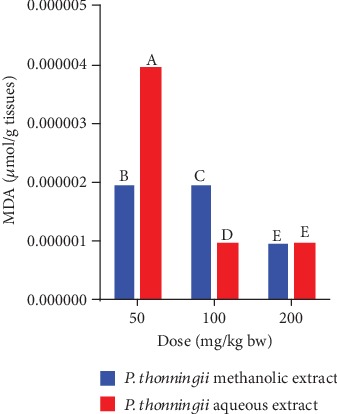
Effects of the aqueous and methanolic stem bark extracts of *P. thonningii* on MDA profile. Bars with the same letter within the same dose level are not significantly different (*p* > 0.05) while those with different letters are significantly different (*p* < 0.05) (unpaired Student's *t*-test).

A comparison of the effects of the aqueous and methanolic stem bark extracts of *P. thonningii* on MDA profiles in the brains of cognitive-impaired mice models was also done in this study. At a dose level of 50 mg/kg bw, the experimental mice that received the methanolic extract of *P. thonningii* had significantly lower MDA levels in their brains compared with the mice that received the *P. thonningii* aqueous extract at the same dose (*p* < 0.05; Figure 12). However, at a dose of 100 mg/kg bw, the experimental mice treated with the aqueous stem bark extract of *P. thonningii* had a significantly lower MDA profile in their brains compared with the mice that received the methanolic stem bark extract of this plant at the same dose (*p* < 0.05; Figure 12). There were no significant differences in MDA profiles in the brains of experimental mice that were treated with a dose of 200 mg/kg bw of the aqueous and methanolic stem bark extracts of *P. thonningii* (*p* > 0.05; Figure 12).

### 3.4. Qualitative Phytochemical Screening of the Aqueous and Methanolic Stem Bark Extracts of *P. thonningii*

Upon qualitative phytochemical screening of the aqueous and methanolic stem bark extracts of *P. thonningii*, it was observed that anthracene glycosides and terpenoids were absent in all the studied plant extracts (Table 5). However, cardenolide glycosides, coumarins, phenols, steroids, saponins, and flavonoids were present in the aqueous and methanolic stem bark extracts of *P. thonningii* (Table 5).

## 4. Discussion

Plants have been utilized since antiquity as sources of food and medicines among other uses [34]. They are a valuable source of various biologically active molecules that offer valuable leads for new drug discovery and development. Proper phytochemical handling and processing of medicinal plant materials is imperative to optimize the concentration and assure integrity of bioactivities of the constituents [36].

Research has shown that aqueous and methanolic extracts of plants mainly constitute antioxidant phytochemicals, and the percentage yields are dependent on the concentration of these compounds in the plant material. In addition, higher percentage yields are indicative of higher extractive value of the solvent used and, consequently, higher concentration of extracted phytocompounds [37]. Therefore, the high percentage yield obtained for the methanolic stem bark extract of *P. thonningii* depicts high concentration of phytochemical compounds, more particularly the antioxidant compounds soluble in the used solvents.

Dementia of the Alzheimer's type is the commonest neurodegenerative disease characterized by progressive impairment of the brain which causes its imminent dysfunction [1]. It is now well understood that the symptoms associated with brain dysfunction, especially cognitive impairment, result from damage of cholinergic neurons in the central cholinergic system. The central cholinergic system plays a central role in learning, intelligence, reasoning, judgement, and memory functions of the brain [8, 38]. Therefore, a disruption of the central cholinergic system, their neurons, or the signaling and proper communication triggers pathogenesis and progression of AD-associated cognitive dysfunction [8, 39].

The Morris water maze method adopted in this study to investigate the effects of aqueous and methanolic extracts of *P. thonningii* has been used extensively to evaluate spatial learning and memory in rodents in antidementia studies. It evaluates both the reference and spatial memories in animal models and humans. Moreover, it detects changes in the central cholinergic system, which controls cognitive functions of the brain. In this method, the experimental animals navigate a water maze from a predetermined starting position with the help of local and extramaze visual cues to search for and locate a hidden escape platform [30, 32].

In this study, cognitive impairment was induced in experimental mice via the administration of scopolamine, a drug that hinders memory and cognitive functions by blocking muscarinic receptors in the central and peripheral nervous systems [40–42]. Scopolamine has been utilized widely to evaluate the potential of drug agents proposed for dementia therapy because it causes symptoms synonymous to those caused by neurodegenerative dementias in a short time [39, 43–50].

Spatial learning and memory are majorly dependent on the integrity of the hippocampus. To appraise spatial memory acquisition, retention, and retrieval, monitor parameters including transfer latency, navigation distance, and latency in the platform quadrant are determined. Following the successful acquisition of spatial memory by the experimental animal, it then processes the acquired information and retrieves it later when needed. This is reflected in the experimental animal's ability to memorize and locate the hidden escape platform in the water maze within a short period of time, covering a short distance and spending more time (longer latency) in the platform quadrant [30, 32, 51, 52].

The results of this study showed that scopolamine successfully imparted cognitive deficits in mice as evidenced by the longer transfer latency and navigation distance. This is attributable to the anticholinergic effects of scopolamine in the brains of the subjects, which deter proper cholinergic transmission. The blockade of the central cholinergic system is associated with an impairment of the brain's ability to create, consolidate, retain, and retrieve memory [49].

Previous studies have shown that scopolamine is a potent inducer of oxidative stress (OS), neuroinflammation, and apoptosis of the brain cells [53]. In addition, scopolamine-induced OS causes amnesia by inhibiting proper functioning of endogenous antioxidant mechanisms including the superoxide dismutase (SOD), the glutathione s-transferase, the catalase, and the glutathione peroxidase thus driving forward oxidative damage to neural cells and their components [16, 54–57]. It is therefore, partly, that the aqueous stem bark extract of *P. thonningii* potentially modulated the activity of the endogenous antioxidant enzymes, thereby averting oxidative damage to brain cells and their components.

One of the most susceptible components to oxidative damage in biological systems is the unsaturated fatty acids because they possess unstable electrons near double bonds. Thus, they are sensitive to lipid peroxidation which increases exponentially with an increase in the degree of unsaturation [58]. The brain organ is highly susceptible to oxidative stress, and this has been considered as the driving force in many neurodegenerative conditions and diseases [7, 59]. Due to its high oxygen consumption (~20% of basal O_2_) in adult humans for its aerobic metabolism, the presence of polyunsaturated fatty acids as well as redox-active metals like copper and iron increases the brain's vulnerability to oxidative stress damage [8].

It has been further determined that mitochondrial abnormalities, neurofibrillary tangles (NFTs), soluble amyloid beta-protein (A*β*), A*β* fibrils, and ageing all are attributable to high degrees of free radical oxidative stress [11]. Imbalances in oxidative equilibrium have been linked to initiation and sustenance of neurodegenerative disorders like AD and PD [12].

Lipid peroxidation causes oxidative rancidity of cellular lipids leading to the production of toxic amalgams in the body. It has been identified as one of the major triggers and drivers of various diseases in humans including inflammation and neurologic disorders [60]. Various mechanisms that drive lipid peroxidation have been extensively explored in literature [58, 61–65]. Briefly, lipid peroxidation advances through a free radical cascade that generates various undesirable products, the major ones comprising aldehydes. The most prominent aldehyde product of lipid peroxidation is malondialdehyde (MDA) and has been widely considered as an oxidative damage marker in physiologic systems [66]. During oxidative stress and associated damage, the levels of lipid peroxidation increase leading to high MDA profiles [63, 67, 68].

Research has demonstrated that scopolamine-induced OS increases the levels of malondialdehyde (MDA) [50, 63, 67–69]. In line with the earlier study by Hritcu et al. [70], *ex vivo* determination of MDA levels in the brains of cognitive-impaired mice demonstrated high MDA profile in the negative control. The experimental mouse groups that were treated with the studied plant extracts except at low doses and the standard drug, Donepezil, had low MDA profiles, an indication of successful oxidative stress attenuation and cognitive impairment reversal. However, these findings differ from a previous study by Losuwannarak et al. [71], in which scopolamine did not cause an increase in lipid peroxidation and hence MDA. These differences are attributable in part to the species of experimental mice used, the dose of scopolamine administered, and the experimental conditions at which the study was performed.

Therefore, antioxidant therapy can ameliorate scopolamine-induced cognitive impairment by preventing propagation of oxidative stress and their markers by either scavenging free radicals and bringing to equilibrium the prooxidant-antioxidant homeostasis or by potentiating the activity of endogenous antioxidant systems [8, 71].

In this study, qualitative phytochemical screening of the aqueous and methanolic stem bark extracts of *P. thonningii* revealed the presence of various phytochemical compounds of biological significance. The results revealed that terpenoids and anthraquinones were absent in the studied extracts. However, these findings differ from a similar study conducted by Kwaji et al. [72] where terpenoids and anthraquinones were present in the alcoholic leaf extract of *P. thonningii*. Research has demonstrated that environmental differences in which medicinal plants grow greatly influence their phytochemical profiles because secondary plant metabolites are essentially produced in response to stress as a protective mechanism [16, 42, 73–75].

Plant phenolics have the widest spectrum of pharmacologic bioactivity due to their antioxidant nature [22]. Therefore, the flavonoids, phenols, coumarins, and tannins in the studied plant extracts are thought to have played a crucial role in attenuating oxidative stress both *in vitro* and *in vivo*. It is conceivable that the cognitive impairment-ameliorating effects of the studied plant extracts could in part be attributed to the presence of these antioxidant compounds that properly restored the redox homeostasis and/or protected the cells from OS damage.

## 5. Conclusions and Recommendations

From the obtained results, it was concluded that the aqueous and methanolic stem bark extracts of *P. thonningii* have *in vivo* cognitive-enhancing and *ex vivo* MDA profile-lowering effects on scopolamine-induced cognitively impaired mice models. Furthermore, it was concluded that the studied plant extracts have cognitive-enhancing and antioxidant phytochemicals.

From this study, the studied plant extracts have a promising potential for the management of AD-associated cognitive deficits. Further studies aimed at isolating and characterizing the pure phytoactive principles for cognitive enhancement are recommended. Furthermore, the specific mechanism(s) through which the phytoactive components of the studied plant extracts exert pharmacologic activity should be established. Toxicity studies on the aqueous and methanolic stem bark extracts of *P. thonningii* should be done so as to determine their safety.

## Figures and Tables

**Table 1:** Treatment of randomized mice for *in vivo* cognitive-enhancing activities.

Treatment groups		Treatment
I	Normal control	Normal saline only (10 ml/kg bw; *i.p*)
II	Negative control	Normal saline+scopolamine (1 mg/kg bw; *i.p*)
III	Positive control	Donepezil (1 mg/kg bw; *p.o*)+scopolamine (1 mg/kg bw; *i.p*)
IV	Experimental group 1	Extract (50 mg/kg bw; *p.o*)+scopolamine (1 mg/kg bw; *i.p*)
V	Experimental group 2	Extract (100 mg/kg bw; *p.o*)+scopolamine (1 mg/kg bw; *i.p*)
VI	Experimental group 3	Extract (200 mg/kg bw; *p.o*)+scopolamine (1 mg/kg bw)

All the administered drugs were prepared in normal saline (0.9% NaCl). Extract: aqueous/methanolic stem bark extracts of *P. thonningii*; *p.o*: oral administration; *i.p*: intraperitoneal administration.

**Table 2:** Percentage yields of study plant extracts.

Plant	Percentage (%) yield	
	Methanol extract	Aqueous extract
*P. thonningii*	38.92%	18.27%

**Table 3:** Effects of methanolic and aqueous stem bark extracts of *P. thonningii* on transfer latency during the acquisition period.

Treatment	Transfer latency (s)		
	Day 1	Day 2	Day 3
Normal control	25.77 ± 0.03^e^	16.27 ± 0.03^f^	7.067 ± 0.03^d^
Negative control (normal saline)	42.33 ± 1.77^a^	36.37 ± 1.95^a^	14.33 ± 1.20^a^
Positive (Donepezil 1 mg/kg bw)	34.60 ± 1.82^c^	18.23 ± 0.07^d^	12.10 ± 0.06^b^
Extract (a)			
50 mg/kg bw	41.50 ± 0.82^a^	25.00 ± 0.93^b^	13.33 ± 1.67^a^
100 mg/kg bw	38.07 ± 0.37^b^	21.83 ± 0.89^c^	12.47 ± 1.33^b^
200 mg/kg bw	37.77 ± 1.60^b^	18.27 ± 0.03^d^	8.37 ± 1.43^c^
Extract (b)			
50 mg/kg bw	35.12 ± 0.03^c^	19.77 ± 0.95^d^	12.47 ± 0.23^b^
100 mg/kg bw	34.90 ± 0.30^c^	17.87 ± 0.91^e^	9.03 ± 0.65^c^
200 mg/kg bw	33.60 ± 1.01^d^	17.30 ± 0.94^e^	4.27 ± 0.78^e^

Extract (a): *P. thonningii* methanolic extract; Extract (b): *P. thonningii* aqueous extract. Values are expressed as *x*˜±SEM; Means with the same superscript letter within the same column are not significantly different (*p* > 0.05) while those with different superscript letters are significantly different (*p* < 0.05) (one-way ANOVA followed by Fisher's LSD).

**Table 4:** Effects of methanolic and aqueous stem bark extracts of *P. thonningii* on navigation distance during the acquisition period.

Treatment	Navigation distance (m)		
	Day 1	Day 2	Day 3
Normal control	9.89 ± 0.89^a^	4.12 ± 0.73^b^	1.52 ± 0.20^b^
Negative control (normal saline)	9.69 ± 0.01^a^	5.78 ± 0.00^a^	3.69 ± 0.25^a^
Positive (Donepezil 1 mg/kg bw)	5.18 ± 1.31^e^	1.99 ± 0.59^c^	1.67 ± 0.84^b^
Extract (a)			
50 mg/kg bw	9.81 ± 0.26^a^	5.632 ± 0.11^a^	4.01 ± 0.89^a^
100 mg/kg bw	7.37 ± 0.00^c^	4.39 ± 0.91^b^	3.85 ± 1.03^a^
200 mg/kg bw	6.62 ± 0.73^d^	4.71 ± 0.42^b^	1.35 ± 0.26^b^
Extract (b)			
50 mg/kg bw	6.36 ± 0.36^d^	2.63 ± 0.32^c^	1.21 ± 0.08^b^
100 mg/kg bw	5.61 ± 0.65^e^	2.306 ± 0.21^c^	1.93 ± 0.58^b^
200 mg/kg bw	3.69 ± 0.23^f^	1.65 ± 0.22^c^	1.44 ± 1.09^b^

Extract (a): *P. thonningii* methanolic extract; Extract (b): *P. thonningii* aqueous extract. Values are expressed as *x*˜±SEM; Means with the same superscript letter within the same column are not significantly different (*p* > 0.05) while those with different superscript letters within the same column are significantly different (*p* < 0.05) (one-way ANOVA followed by Fisher's LSD).

**Table 5:** Phytochemical profile of the aqueous and methanolic stem bark extracts of *P. thonningii*.

Phytochemical	*P. thonningii*	
	Methanolic extract	Aqueous extract
Alkaloids	+	+
Cardenolide glycosides	+	+
Anthraquinones	-	-
Coumarins	+	+
Tannins	+	+
Terpenoids	-	-
Phenols	+	+
Steroids	+	+
Saponins	+	+
Flavonoids	+	+

+ = present; - = absent.

## Data Availability

The data used to support the findings of this study are included within the article. Any additional data is available from the authors upon request
